# Tetraptycene derivatives: synthesis, structure and their self-assemblies in solid state†

**DOI:** 10.1039/d5ra00376h

**Published:** 2025-03-17

**Authors:** Bin-Bin Yang, Si-Yuan Wu, Qing-Pu Zhang, Hui Ma, Yu-Ling Sun, Chun Zhang

**Affiliations:** a College of Life Science and Technology, Huazhong University of Science and Technology Wuhan 430074 China chunzhang@hust.edu.cn

## Abstract

As iptycenes of arenes are fused to a bicyclo[2.2.2]octatriene bridgehead system, there are only odd-sequenced iptycene family members, such as triptycene, pentiptycene and heptiptycene. In order to ensure the completeness of the iptycene family sequence, developing even-sequenced iptycene family members is of great significance. The dimer of anthracene derivatives is a class of tricyclo[2.2.2.2]dodetetraene molecules with four separate phenyl rings, which are structurally similar to the iptycene family and herein referred to as “tetraptycene”. In this work, a series of hydroxyl or methoxy-substituted tetraptycene derivatives from the photochemical reactions of anthracene derivatives was reported. These tetraptycene derivatives were characterized using nuclear magnetic resonance (NMR), mass spectrum (MS) and single-crystal X-ray diffraction (SC-XRD). Moreover, their self-assemblies in the solid state were further discussed. Their properties of modifiability, asymmetry, and rigidity indicate their superiority as novel monomers to construct functional material architectures.

## Abstract 2

A series of hydroxyl- or methoxy-substituted tetraptycene derivatives from the photochemical reactions of anthracene derivatives was obtained and characterized. Their self-assemblies in the solid state were further discussed.

## Graphical abstract

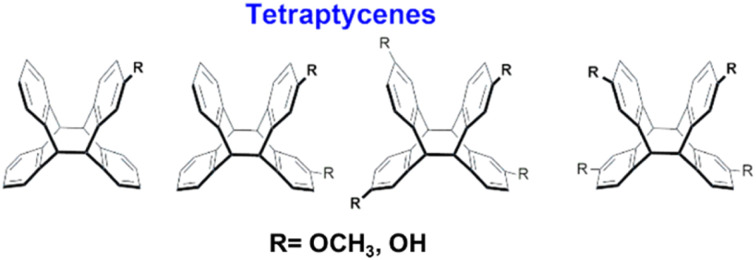

## Introduction

**Scheme 1:**
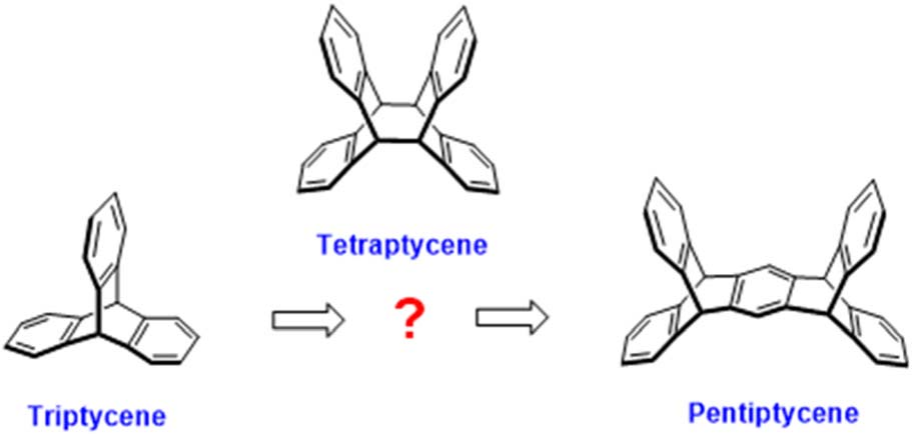
Structure of triptycene, tetraptycene and pentiptycene.

Iptycene chemistry has attracted significant attention because of its potential applications in the fields of supramolecular chemistry,^1^ molecular machines,^2^ and porous materials^3,4^ Hart *et al.* first proposed the concept of “iptycene” on the basis of relevant research on triptycene in 1981.^5^ However, as iptycenes of arenes are fused to a bicyclo[2.2.2]octatriene bridgehead system, the members of the iptycene family are limited to possessing only odd sequences, such as triptycene,^6^ pentiptycene^7^ and heptiptycene (Scheme 1).^8^ Thus, developing even-sequenced members of the iptycene family is of great significance for enriching iptycene chemistry and discovering new material building units.^9^

Anthracene dimer obtained *via* the [4 + 4] photo-induced cycloaddition of anthracene^10^ is a bridged ring compound consisting of a tricyclo[2.2.2.2]dodetetraene fragment linking four independent benzene ring units. Its structure is conformed to the definition of “iptycene” and is therefore referred to as “tetraptycene” (Scheme 1). It exhibits significant advantages in the construction of porous polymers owing to its unique geometric structure, molecular rigidity, and excellent photoreversibility. Meanwhile, its industrially scalable and environmentally friendly synthesis process endows it with outstanding potential for industrial applications. Recently, tetraptycene derivatives are being used as precursors or intermediates for the research on molecular machines,^11^ supramolecular microreactors,^12^ nanorings,^13^ hydrogels^14^ and other advanced materials owing to their special properties.^15^ Furthermore, tetraptycene derivatives with different quantities and types of groups can be used as the basic building monomers of functional materials, which can be conveniently obtained *via* the [4 + 4] photodimerization of the modified anthracene.^16^ For example, Inoue *et al.* have synthesized a variety of highly enantioselective dicarboxylic substituted tetraptycene with 2-anthracenecarboxylic acid as the precursor.^17^ Cong *et al.* have reported different tetrasubstituted stereoisomers with 2,6-dibromoanthrene as the raw material, whose halogen groups were substituted by tetraborate groups through the Miyaura borylation reaction after dimerization, which provided active sites for the subsequent construction of nanohoops.^18^ However, scarcity of the tetraptycene species, places restrictions on their extensive applications to a considerable extent.^19^

Introducing a hydroxyl group (a well-known group for improving polarity) into the tetraptycene molecule, followed by reacting it with acyl chlorides, carboxyl groups and other groups and converting it into specific functional groups, can further facilitate the structural studies and applications of tetraptycene.^20^ Herein, we used anthracene, 2,6-dimethoxyanthracene, and 2-hydroxyanthracene as raw materials to synthesize a series of methoxyl or hydroxyl group-substituted tetraptycene under an irradiation of a 320 nm light source. The regioisomers and structures were identified using ^1^H NMR, ^13^C NMR, mass spectrometry, and single-crystal X-ray diffraction.

## Result and discussion

### Synthesis of methoxy substituted tetraptycene

**Scheme 2:**
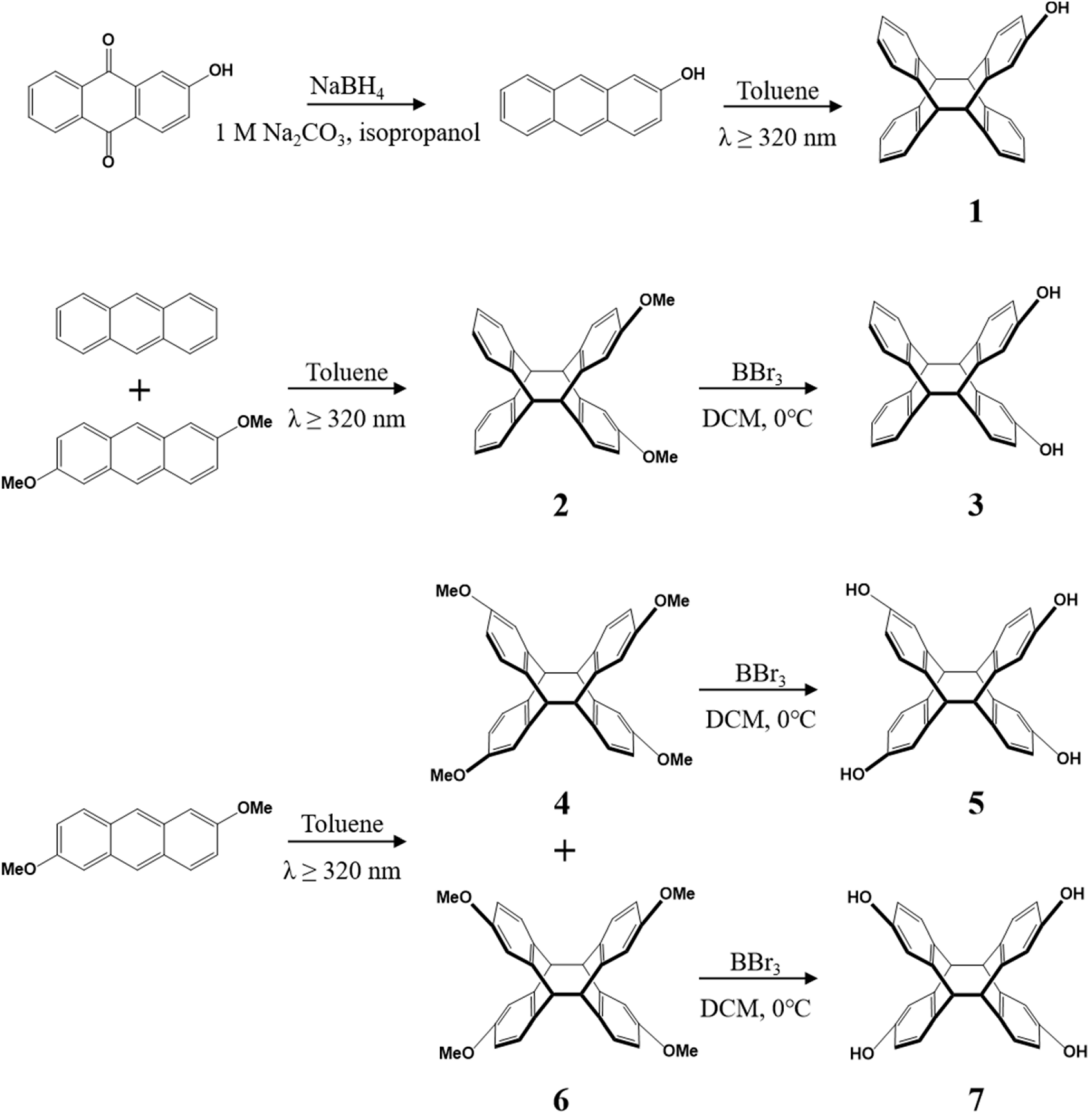
Synthesis of tetraptycenes.

As a photosensitive molecule with high reactivity, anthracene will undergo photo-induced cycloaddition reaction when exposed to ultraviolet light (*λ* > 300 nm), which coordinates the rearrangement of molecular bonds between the two anthracene molecules, and then the dimeric structure formed by the [4 + 4] cycloaddition reaction. On the basis of the light-mediated chemistry of anthracene, which has been studied in great detail, we synthesized compound 2 under 320 nm UV illumination using 5 equiv. of anthracene and 1 equiv. of 2,6-dimethoxyanthracene as raw materials with toluene as solvent, which gives 73% yield of dimethoxy tetraptycene 2 (Scheme 2). The utilization of excessive anthracene was to reduce the self-polymerization of 2,6-dimethoxyanthracene. The reaction of 2,6-dimethoxyanthracene toluene solution was irradiated by 320 nm UV in an argon atmosphere, and compounds 4 and 6 could be obtained in 53 and 36% yields, respectively (Scheme 2). It was noteworthy that tetramethoxy isomers 4 and 6 share different solubilities, from which compound 4 dissolved in toluene while compound 6 precipitated from toluene as a white solid at the end of the reaction.

### Functional transformation of hydroxy substituted tetraptycene

Using compounds 2, 4 and 6 as precursors and boron tribromide as the catalyst, the dihydroxy and tetrahydroxy substituted tetraptycenes (compounds 3, 5 and 7) with improved solubility were gained *via* the demethylation reaction, which gives 69%, 68% and 92% yields, respectively (Scheme 2). In particular, as an extremely poorly soluble white solid produced in the photo-polymerization reaction, compound 6 converted to tetrahydroxy substituted tetraptycene 7 after demethylation, which was soluble in acetone and tetrahydrofuran. We synthesized not only disubstituted and tetrasubstituted tetraptycene derivatives, but also mono-substituted 2-hydroxytetraptycene 1 from 2-hydroxyanthracene (Fig. S7†). The orange solid 2-hydroxyanthracene was synthesized from 2-hydroxyanthraquinone with 10 equiv. NaBH_4_ and 1 M Na_2_CO_3_ in isopropanol and obtained in a 54% yield. The 2-hydroxyanthracene was dissolved in toluene with 5 equiv. of anthracene under a 320 nm wavelength light source irradiation to produce 1. The excessive amount of anthracene was utilized to prevent the self-polymerization of 2-hydroxyanthracene. The white solid 1 exhibited good solubility in toluene and ethyl acetate with a 35% yield.

### Chemical structures of tetraptycene derivatives

The structure of methoxy-substituted tetraptycene could be easily confirmed by ^1^H NMR, ^13^C NMR, MS and FT-IR data. Compared with the ^1^H NMR spectrum of tetraptycene that we prepared according to ref. 21, the corresponding chemical shifts of the hydrogen atoms on bridgehead carbons and aromatic rings of compounds 2 and 4 were both shifted upfield, which was caused possibly by the inductive effect due to the modified methoxy group. The strong coupling effect between the hydrogen atoms on the bridgehead carbon of compound 2 resulted in peak distortion when chemical shift occurred, leading to an increase in the internal peak integrals and a decrease in the external peak integrals (Fig. S2a†). In addition, the characteristic peak corresponding to the methoxy group appeared at 3.67 ppm in the spectrum of compounds 2 and 4 (Fig. S4a†). In the ^13^C NMR spectrum, twelve carbon signals could be found in compound 2 and eight carbon signals in compound 4, corresponding to their structures, respectively. Compared with the bridgehead carbon of compound 4 (53.08 ppm), compound 2 showed two splits for their bridgehead carbon signals at 53.38 and 53.06 due to the effect of different qualities and distributions of methoxy groups. Moreover, the MS data also implied the successful synthesis of compounds 2 and 4 (Fig. S10†). For the FI-IR spectra of methoxy-substituted tetraptycene derivatives, as shown in Fig. S8,† all three compounds 2, 4 and 6 had stretching vibration signals at 2830–2820 cm^−1^, corresponding to the methoxy group on the benzene ring. Also, the stretching vibration signals of the C–H bonds on the bridgehead carbon appeared at 3000–2900 cm^−1^. The peak signals of compound 6 similar to those of 2,2′,6,6′-tetramethoxytetraptycene 4 further illustrated the similarity of their molecular structures. Furthermore, the ultraviolet-visible spectra (Fig. S15a†) and fluorescence emission spectra (Fig. S15b†) of compound 4 and 2,6-dimethoxyanthracene exhibit significant differences. Especially in the fluorescence emission spectra, 2,6-dimethoxyanthracene displays two prominent peaks at 440 nm. However, after the [4 + 4] cycloaddition reaction, the dimer product 2,2′,6,6′-tetramethoxytetraptycene 4 exhibits no fluorescence emission in the same region.

The structures of hydroxy-substituted tetraptycenes 1, 3, 5 and 7 were verified by ^1^H NMR, ^13^C NMR, MS and FT-IR. For 2,6-dihydroxytetraptycene 3 (Fig. S3†), the signal peak of the hydroxyl group appeared at 8.81 ppm concomitantly with the disappearance of the 3.67 ppm peak corresponding to the hydrogen atom on the methoxy group in the ^1^H NMR spectrum. Besides, the ^13^C NMR spectrum showed twelve aromatic carbons and two bridgehead carbons signals, confirming the structure of compound 3. The ^1^H NMR spectrum analysis of compounds 5 and 7 showed that the corresponding peak positions and peak integrals of hydrogen on the benzene ring and bridgehead carbon were the same, and neither of them had the signal of the methoxy group (Fig. S5 and S6†). Moreover, the ^13^C NMR spectrum and molecular weight of the two compounds were identical, which helped to speculate that compounds 5 and 7 were the isomers of tetrahydroxy tetraptycene. As for compound 1, the signal peak of the hydroxyl group, in which the peak area ratio was half that of dihydroxytetraptycene 3, was likewise present at 8.83 ppm in the ^1^H NMR spectrum. Expectedly, it clearly showed the signals of four bridgehead carbons (52.21, 52.94, 53.08, 53.18 ppm) in the ^13^C NMR spectrum (Fig. S1b†). The characterization results of the MS spectra also supported the successful preparation of the hydroxy-substituted tetraptycenes (Fig. S11–S14†). The FI-IR spectra (Fig. S8†) of the hydroxyl-substituted tetraptycene derivatives showed that the stretching vibration signals of the hydroxyl groups on benzene rings were at 3500 cm^−1^ and 3050 cm^−1^. In Fig. S9,† due to the obvious water signal, the hydroxyl signal was almost obscured, and only a weak signal peak can be observed at 3050 cm^−1^. It was assumed that the water absorption of tetrasubstituted hydroxyl samples was stronger than that of the disubstituted and monosubstituted samples, which absorbed more water in the air.

### Solid structures and self-assemblies of tetraptycene derivatives

**Fig. 1:**
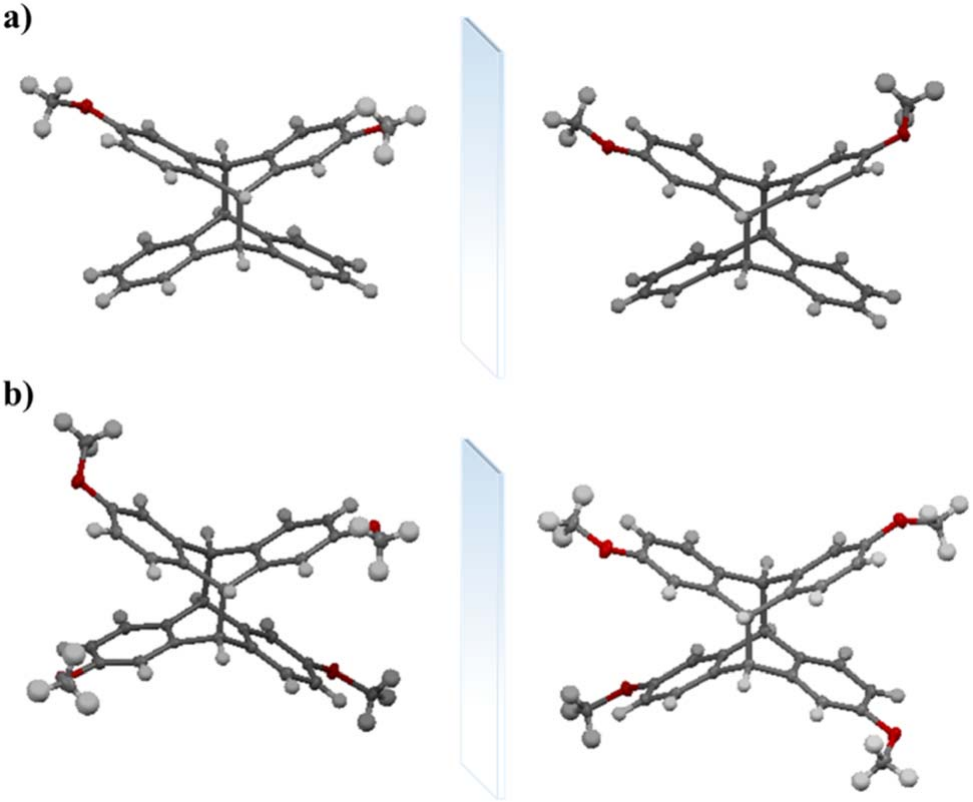
Enantiomeric structure of 2,6-dimethoxytetraptycene 2 (a) and 2,2′,6,6′-tetramethoxytetraptycene 4 (b) obtained from the single crystal data.

**Fig. 2:**
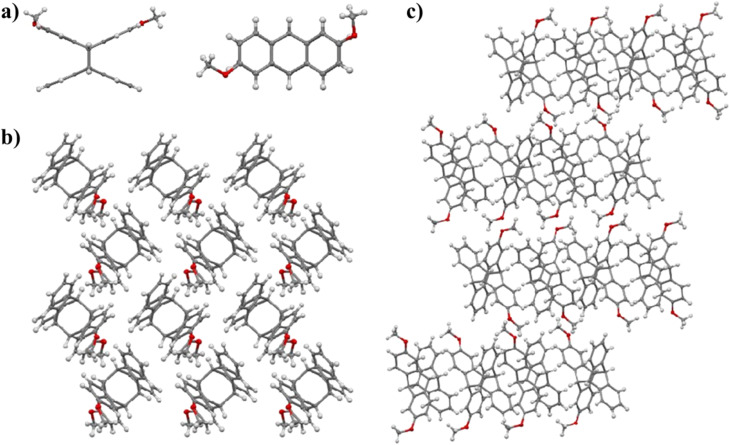
(a) Two views of the crystal structure of 2,6-dimethoxytetraptycene. Packing of 2,6-dimethoxytetraptycene on the *bc* plane (b) and layer by layer stacking along the *a*-axis (c).

**Fig. 3:**
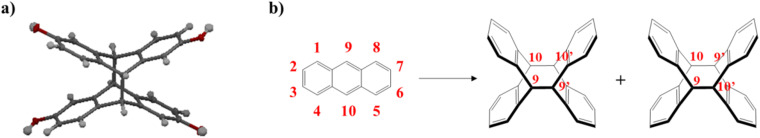
(a) Crystal structure of compound 7. (b) Random rotation and arrangement of anthracene molecules in the photoreaction.

We determined the structures of 2,6-dimethoxytetraptycene 2 and 2,2′,6,6′-tetramethoxytetraptycene 4 using single-crystal X-ray diffraction of the corresponding single crystals, which were obtained by the slow evaporation of dichloromethane. As shown in Fig. 1, the enantiomers with opposite configurations existed in the cells of racemic compounds 2 and 4, which were cross-stacked in space. Especially, from the single crystal data of compound 2, we could see that the enantiomers were staggered on the *bc* plane and stacked layer by layer along the *a*-axis, as shown in Fig. 2. Thus, the above conjecture that compounds 5 and 7 were isomers was confirmed *via* the single-crystal X-ray diffraction analysis of compound 7 (Fig. 3a), from which we inferred the molecular structure of the insoluble compound 6. We believe that during the photoreaction of dimethoxyanthracene, the molecule was randomly rotated and aligned as shown in Fig. 3b, resulting in the production of isomeric compounds 4 and 6 with equal probability, which was further explained by the similar actual yields of approximately 50%. Afterwards, the MS spectra and single-crystal X-ray diffraction provided a more definitive characterization of the structure of compound 1. Based on the data from single crystal diffraction, the angle between the two benzene rings of tetraptycene was approximately 130°. However, the magnitudes of the angles varied slightly because of the variety, quantity and position of the substituents, which caused the distortion of the phenyl ring to varying degrees. The four angles of compounds 1 (132.75°, 134.37°, 132.77°, 134.12°), 2 (130.44°, 131.82°, 132.64°, 133.28°), 4 (Angles of the two enantiomers: 128.23°, 129.57°, 129.28°, 130.46°, 128.13°, 132.25°, 131.33°, 132.60°), 7 (132.69°, 132.85°, 132.69°, 132.85°) were convincing examples, respectively. The precise angle between the phenyl rings was certainly an aid to the calculation of theoretical models that use tetraptycene derivatives as monomers.

**Fig. 4:**
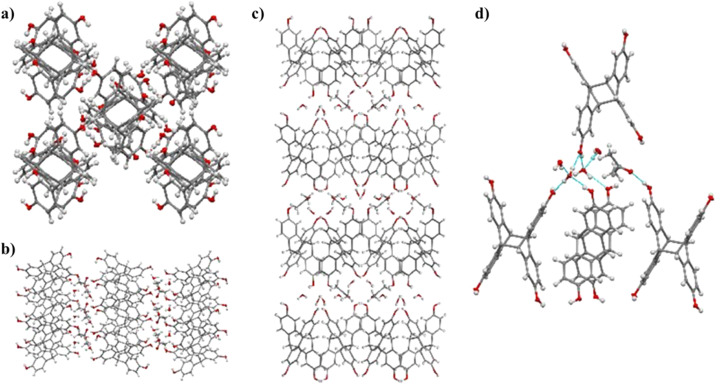
Packing of 2,2′,6,6′-tetrahydroxytetraptycenes 7 along axes *a* (a), *b* (b) and *c* (c). (d) Hydrogen bonding between the solvent molecule and 2,2′,6,6′-tetrahydroxytetraptycenes 7.

Finally, we investigated the solid-state self-assemblies of the hydroxyl-substituted tetraptycene derivatives. Three water molecules and one acetone molecule were found in the crystal data of 2,2′,6,6′-tetrahydroxytetraptycenes 7, which coincided with the chemical formula and molecular weight obtained from the crystal data. Acetone was intimately and orderly involved in the packing of crystals with water molecules due to the strong interaction between the solvent molecules and the phenolic hydroxyl group in 2,2′,6,6′-tetrahydroxytetraptycenes 7. The distribution position of agents was related to the hydrophilicity and hydrophobicity of the lamellar structures, with the methyl group of acetone positioned between the aromatic rings far from the hydroxyl group and the water molecules closer to the hydroxyl group (Fig. 4). Analysis of the hydrogen bonding between 2,2′,6,6′-tetrahydroxytetraptycenes 7 and solvent molecules revealed that there was no hydrogen bonding between the water molecules and acetone or between the phenolic hydroxyl groups of two molecules of 7, while compound 7 had strong hydrogen bonding interactions with water molecules and acetone. As shown in Fig. 4d, three hydrogen bonds (*d*_H⋯O_ = 1.945 Å, 1.940 Å, 1.884 Å, *θ*_O–H⋯O_ = 173.49°, 174.27°, 170.86°) existed between a water molecule and 2,2′,6,6′-tetrahydroxytetraptycenes 7. There was also a hydrogen bond (*d*_H⋯O_ = 1.998 Å, *θ*_O–H⋯O_ = 162.97°) between the two water molecules. One acetone molecule had only one hydrogen bond (*d*_H⋯O_ = 1.902 Å, *θ*_O–H⋯O_ = 174.24°) with tetrahydroxytetraptycene 7.

**Fig. 5:**
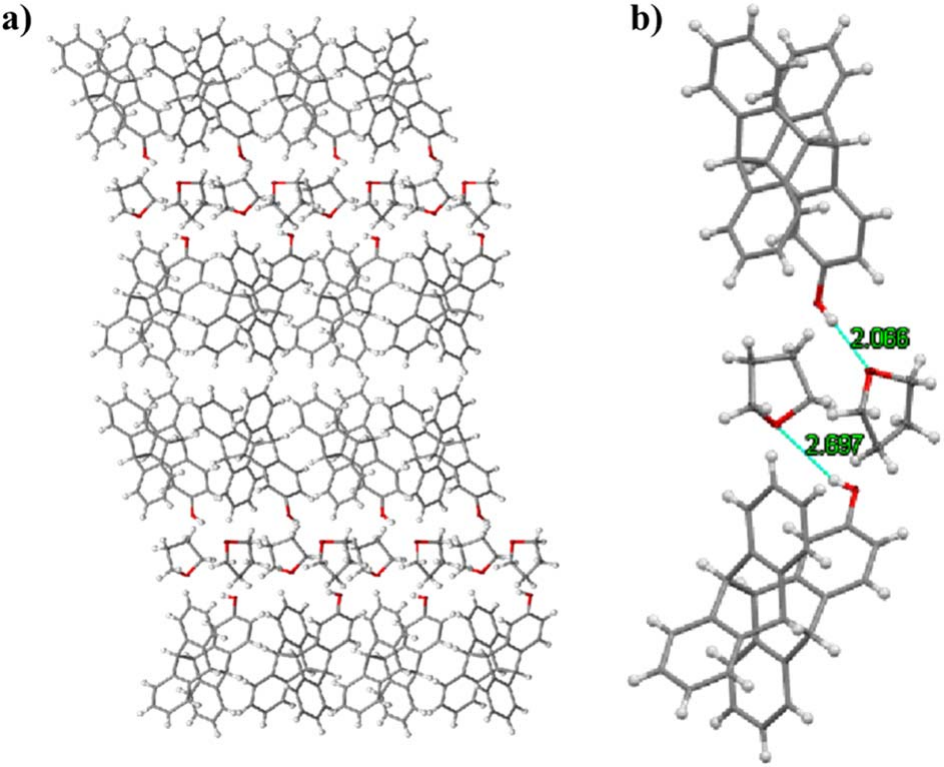
(a) Packing of 2-hydroxytetraptycene 1 along the *b* axis. (b) Hydrogen bonding between tetrahydrofuran and 2-hydroxytetraptycene 1.

We also analyzed the distribution of tetrahydrofuran as a solvent in the 2-hydroxytetraptycene 1 crystal and found that the tetrahydrofuran molecules with opposite orientations were staggered between the layers with hydroxyl groups, which formed two hydrogen bonds (*d*_H⋯O_ = 2.066 Å, 2.267 Å, *θ*_O–H⋯O_ = 145.72°, 138.50°) with 2-hydroxytetraptycene 1 (Fig. 5). The hydrogen bonds between the host and the solvent molecule, as the guest, might play an important role in the crystal accumulation of the tetraptycene molecule.

## Conclusions

For the first time, we reported the synthesis and exact structures of a series of methoxy and hydroxyl-substituted tetraptycene derivatives using the photochemical reactions, which have huge potential for applications in molecular machinery, chiral separation, porous materials and other fields due to the three-dimensional rigidity, asymmetry, light reversibility, and multisite modifiability.

## Experimental section

### Materials

All reagents were purchased from commercial suppliers and used without further purification, except for 2,6-dimethoxyanthracene obtained from the Institute of Chemistry, Chinese Academy of Sciences.

### Characterization

The ^1^H NMR spectra and ^13^C NMR spectra were recorded on DMX400 NMR and DMX600 NMR. The Fourier transform infrared (FT-IR) spectra were recorded on a Bruker model VERTEX 70 infrared spectrometer. The FT-MS mass spectra were obtained by a Bruker SolariX 7.0T mass spectrometer. The MALDI-TOF mass spectra were obtained on a BIFLEX III mass spectrometer. The single-crystal data were obtained using an XtaLAB PRO MM007HF single-crystal X-ray diffractometer.

### Synthesis of methoxy-substituted tetraptycene

#### 2,6-Dimethoxytetraptycene (2)

Anthracene (450 mg, 2.5 mmol) and 2,6-dimethoxyanthracene (120 mg, 0.5 mmol) were added to a Schlenk tube, and then toluene (100 mL) as solvent was added. After stirring and dissolving, the reaction system was irradiated with a 320 nm wavelength light source in an argon atmosphere for 24 h. The crude product was obtained by rotary evaporation of the filtrate, which was purified by flash column chromatography on a silica gel (eluant : petroleum ether/CH_2_Cl_2_ = 1 : 1) to give the product (152 mg, 73% yield) as a white solid. ^1^H NMR (600 MHz, CDCl_3_): *δ*3.67 (s, 6H), 4.46 (d, *J* = 11.1 Hz, 2H), 4.53 (d, *J* = 11.1 Hz, 2H), 6.34 (dd, *J* = 8.1, 2.6 Hz, 2H), 6.53 (d, *J* = 2.6 Hz, 4H), 6.81 (d, *J* = 8.1 Hz, 2H), 6.83–6.85 (m, 4H), 6.92–6.95 (m, 4H). ^13^C NMR (151 MHz, CDCl_3_): *δ*53.1, 53.6, 53.7, 55.3, 109.7, 113.5, 125.5, 126.9, 127.1, 127.2, 127.7, 135.7, 143.5, 143.6, 143.7, 145.4, 157.5. HRMS calcd for C_30_H_24_NaO_2_: [M + Na]^+^ 439.1674. Found: 439.2776. Crystallographic data for 2: *M*_r_ = 416.49, monoclinic, space group *P*2_1_/*c*, *a* = 22.5743(3) Å, *b* = 8.02250(10) Å, *c* = 11.7565(2) Å, *α* = 90°, *β* = 101.648(2)°, *γ* = 90°, *V* = 2085.28(5) Å^3^, *Z* = 4, *ρ*_calcd_ = 1.327 g cm^−3^, *μ* = 0.637 mm^−1^, reflections collected 48 372, data/restraints/parameters 4205/0/293, GOF on *F*^2^ 1.237, final *R*_1_ = 0.0682, w*R*_2_ = 0.2089, *R* indices (all data): *R*_1_ = 0.0703, w*R*_2_ = 0.2101, largest diff. peak and hole: 0.44 and −0.32 e Å^−3^, CCDC-2376824.

#### 2,2′,6,6′-Tetramethoxytetraptycene (4 and 6)

2,6-Dimethoxyanthracene (800 mg, 3.35 mmol) was added to a Schlenk tube, and then toluene (150 mL) as solvent was added. After stirring and dissolving, the reaction system was irradiated with a 320 nm wavelength light source in an argon atmosphere for 24 h. The white precipitate from the reaction was washed 2–3 times with toluene and CH_2_Cl_2_, and then dried in vacuum to give 2,2′,6,6′-tetramethoxytetraptycene 6 (431 mg, 53% yield). Besides, the pale yellow crude product was obtained by rotary evaporation of the filtrate, which was purified by flash column chromatography on silica gel (eluant : petroleum ether/CH_2_Cl_2_ = 1 : 2) to give the 2,2′,6,6′-tetramethoxytetraptycene 4 (290 mg, 36% yield) as a white solid. ^1^H NMR (400 MHz, CDCl_3_): *δ*3.67 (s, 12H), 4.42 (s, 4H), 6.34 (dd, *J* = 8.1, 2.6 Hz, 4H), 6.51 (d, *J* = 2.6 Hz, 2H), 6.81 (d, *J* = 8.1 Hz, 2H), 6.83–6.86 (m, 4H), 6.92–6.95 (m, 4H). ^13^C NMR (101 MHz, CDCl_3_): *δ*53.1, 55.3, 109.7, 113.5, 127.8, 135.7, 145.4, 157.4. HRMS calcd for C_32_H_28_NaO_4_: [M + Na]^+^ 499.1885. Found: 499.3360. Crystallographic data for 4: *M*_r_ = 476.54, monoclinic, space group *C*2/*c*, *a* = 29.0799(2) Å, *b* = 14.40550(10) Å, *c* = 22.9347(2) Å, *α* = 90°, *β* = 92.9710(10)°, *γ* = 90°, *V* = 9594.67(13) Å^3^, *Z* = 16, *ρ*_calcd_ = 1.320 g cm^−3^, *μ* = 0.686 mm^−1^, reflections collected 51 814, data/restraints/parameters 9603/0/657, GOF on *F*^2^ 1.044, final *R*_1_ = 0.0405, w*R*_2_ = 0.1059, *R* indices (all data): *R*_1_ = 0.0457, w*R*_2_ = 0.1099, largest diff. peak and hole: 0.23 and −0.22 e Å^−3^, CCDC-2376825.

### Synthesis of hydroxy-substituted tetraptycene

#### 2-Hydroxyanthracene

A 1 M solution (120 mL) of Na_2_CO_3_ dissolved with NaBH_4_ (3.38 g, 90 mmol) was poured into a round bottom flask with 25 mL isopropanol. After raising the temperature to the boiling point, the Na_2_CO_3_ solution (40 mL) dissolved with 2-hydroxyanthraquinone (2 g, 9 mmol) was added dropwise under vigorous stirring. The mixture was stirred continuously for 20 minutes under the heating condition and then cooled to room temperature. After acidification with 3 M HCl, filtration, extraction with ethyl acetate, and drying with anhydrous Na_2_SO_4_, the concentrated organic phase was separated by flash column chromatography on silica gel (eluant : petroleum ether/ethyl acetate = 3 : 1). Finally, the orange solid (940 mg, 54% yield) is obtained.

#### 2-Hydroxytetraptycene (1)

Anthracene (900 mg, 5 mmol) and 2-hydroxyanthracene (190 mg, 1 mmol) were added to a Schlenk tube, and then toluene (150 mL) as solvent was added. After stirring and dissolving, the reaction system was irradiated with a 320 nm wavelength light source in an argon atmosphere for 24 h. The crude product was obtained by rotary evaporation of the filtrate, which was purified by flash column chromatography on silica gel (eluant : petroleum ether/ethyl acetate = 5 : 1) to give the product (126 mg, 35% yield) as a white solid. ^1^H NMR (600 MHz, DMSO-d_6_): *δ*4.48 (d, *J* = 8.8 Hz, 1H), 4.50 (d, *J* = 7.9 Hz, 1H), 4.55–4.59 (m, 2H), 6.14 (dd, *J* = 7.9, 2.4 Hz, 1H), 6.40 (d, *J* = 2.4 Hz, 1H), 6.69 (d, *J* = 7.9 Hz, 1H), 6.75–6.77 (m, 4H), 6.81–6.82 (m, 2H), 6.89–6.98 (m, 6H), 8.83 (s, 1H). ^13^C NMR (151 MHz, DMSO-d_6_): *δ*52.2, 52.9, 53.1, 53.2, 111.8, 114.8, 125.4, 125.5, 125.6, 127.0, 127.2, 127.4, 127.9, 134.5, 144.0, 144.1, 144.1, 144.5, 144.5, 144.7, 145.3, 155.0. HRMS: calcd for C_28_H_20_ClO: [M + Cl]^+^ 372.1514. Found: 407.1210. Crystallographic data for 1: *M*_r_ = 148.98, monoclinic, space group *Pc*, *a* = 23.7924(9) Å, *b* = 8.0783(2) Å, *c* = 11.8488(3) Å, *α* = 90°, *β* = 97.077(3)°, *γ* = 90°, *V* = 2260.02(12) Å^3^, *Z* = 10, *ρ*_calcd_ = 1.095 g cm^−3^, *μ* = 0.502 mm^−1^, reflections collected 22 343, data/restraints/parameters 6684/1/524, GOF on *F*^2^ 1.136, final *R*_1_ = 0.1021, w*R*_2_ = 0.2610, *R* indices (all data): *R*_1_ = 0.1129, w*R*_2_ = 0.2746, largest diff. peak and hole: 0.65 and −0.37 e Å^−3^, CCDC-2376823.

#### 2,6-Dihydroxytetraptycene (3)

2,6-Dimethoxytetraptycene 2 (320 mg, 0.77 mmol) was dissolved in CH_2_Cl_2_ and then 0.35 mL BBr_3_ was slowly dripped in at 0 °C. After 12 h of stirring at room temperature, water was added to quench the reaction. A light green solid gained by centrifugation and the removal of the supernatant was washed 2–3 times with ultrapure water, CH_2_Cl_2_, ethyl acetate, and dried in vacuum to give the product (207 mg, 69% yield). ^1^H NMR (600 MHz, DMSO-d_6_): *δ*4.36 (d, *J* = 11.1 Hz, 2H), 4.52 (d, *J* = 11.0 Hz, 2H), 6.12 (dd, *J* = 7.9, 2.4 Hz, 2H), 6.36 (d, *J* = 2.3 Hz, 2H), 6.65 (d, *J* = 8.0 Hz, 2H), 6.79–6.80 (m, 4H), 6.90–6.95 (m, 4H), 8.81 (s, 2H). ^13^C NMR (101 MHz, DMSO-d_6_): *δ*52.3, 53.2, 111.5, 114.5, 125.4, 125.5, 127.1, 127.3, 127.8, 134.4, 144.4, 144.5, 145.9, 155.0. HRMS calcd for C_28_H_20_ClO_2_: [M + Cl]^+^ 423.1152. Found: 423.1150.

#### 2,2′,6,6′-Tetrahydroxytetraptycene (5)

2,2′,6,6′-Tetramethoxytetraptycene 4 (100 mg, 0.21 mmol) was dissolved in CH_2_Cl_2_ and then 0.22 mL BBr_3_ was slowly dripped in at 0 °C. After 12 h of stirring at room temperature, water was added to quench the reaction. A light green solid gained by centrifugation and the removal of supernatant was washed 2–3 times with ultrapure water, CH_2_Cl_2_, ethyl acetate, and dried with vacuum to give the product 5 (60 mg, 68% yield). ^1^H NMR (600 MHz, DMSO-d_6_): *δ*4.26 (s, 4H), 6.13 (dd, *J* = 7.9, 2.4 Hz, 4H), 6.32 (d, *J* = 2.4 Hz, 4H), 6.65 (d, *J* = 7.9 Hz, 4H), 8.79 (s, 4H). ^13^C NMR (101 MHz, DMSO-d_6_): *δ*52.5, 111.3, 114.5, 127.8, 134.7, 146.3, 154.8. HRMS: calcd for C_28_H_20_O_4_: [M]^+^ 420.1362. Found: 420.1323.

#### 2,2′,6,6′-Tetrahydroxytetraptycene (7)

2,2′,6,6′-Tetramethoxytetraptycene 6 (200 mg, 0.42 mmol) was dissolved in CH_2_Cl_2_ and then 0.45 mL BBr_3_ was slowly dripped in at 0 °C. After 12 h of stirring at room temperature, water was added to quench the reaction. A light green solid gained by centrifugation and the removal of supernatant was washed 2–3 times with ultrapure water, CH_2_Cl_2_, ethyl acetate, and dried with vacuum to give the product 7 (162 mg, 92% yield). ^1^H NMR (400 MHz, DMSO-d_6_): *δ*4.26 (s, 4H), 6.12 (dd, *J* = 7.9, 2.4 Hz, 4H), 6.35 (d, *J* = 2.4 Hz, 4H), 6.61 (d, *J* = 8.0 Hz, 4H). ^13^C NMR (151 MHz, DMSO-d_6_): *δ*52.6, 111.4, 114.6, 127.6, 134.9, 146.2, 154.7. HRMS calcd for C_28_H_20_ClO_4_: [M + Cl]^+^ 455.1050. Found: 455.1050. Crystallographic data for 7: *M*_r_ = 532.56, monoclinic, space group *P*2_1_/*c*, *a* = 26.1553(2) Å, *b* = 11.01180(10) Å, *c* = 8.96460(10) Å, *α* = 90°, *β* = 91.5810(10)°, *γ* = 90°, *V* = 2580.97(4) Å^3^, *Z* = 4, *ρ*_calcd_ = 1.371 g cm^−3^, *μ* = 0.812 mm^−1^, reflections collected 59 598, data/restraints/parameters 5207/0/367, GOF on *F*^2^ 1.046, final *R*_1_ = 0.0418, w*R*_2_ = 0.1102, *R* indices (all data): *R*_1_ = 0.0431, w*R*_2_ = 0.1113, largest diff. peak and hole: 0.40 and −0.37 e Å^−3^, CCDC-2376826.

## Data availability

The data underlying this study are available in the published article and its ESI.†

## Conflicts of interest

The authors declare that they have no known competing financial interests or personal relationships that could have appeared to influence the work reported in this paper.

## Supplementary Material

RA-015-D5RA00376H-s001

RA-015-D5RA00376H-s002
