# Supplementary material for: Tetraptycene derivatives: synthesis, structure and their self-assemblies in solid state
Source: RSC Adv. 2025 Mar 17;15(11):8293–9. doi: 10.1039/d5ra00376h (PMC11912553; doi:10.1039/d5ra00376h)
Supplement: RA-015-D5RA00376H-s001 [file RA-015-D5RA00376H-s001.pdf]

Supplementary Information (SI) for RSC Advances.
This journal is © The Royal Society of Chemistry 2025

Supporting information

Tetraptycene Derivatives: Synthesis, Structure and their Self-assemblies in Solid State

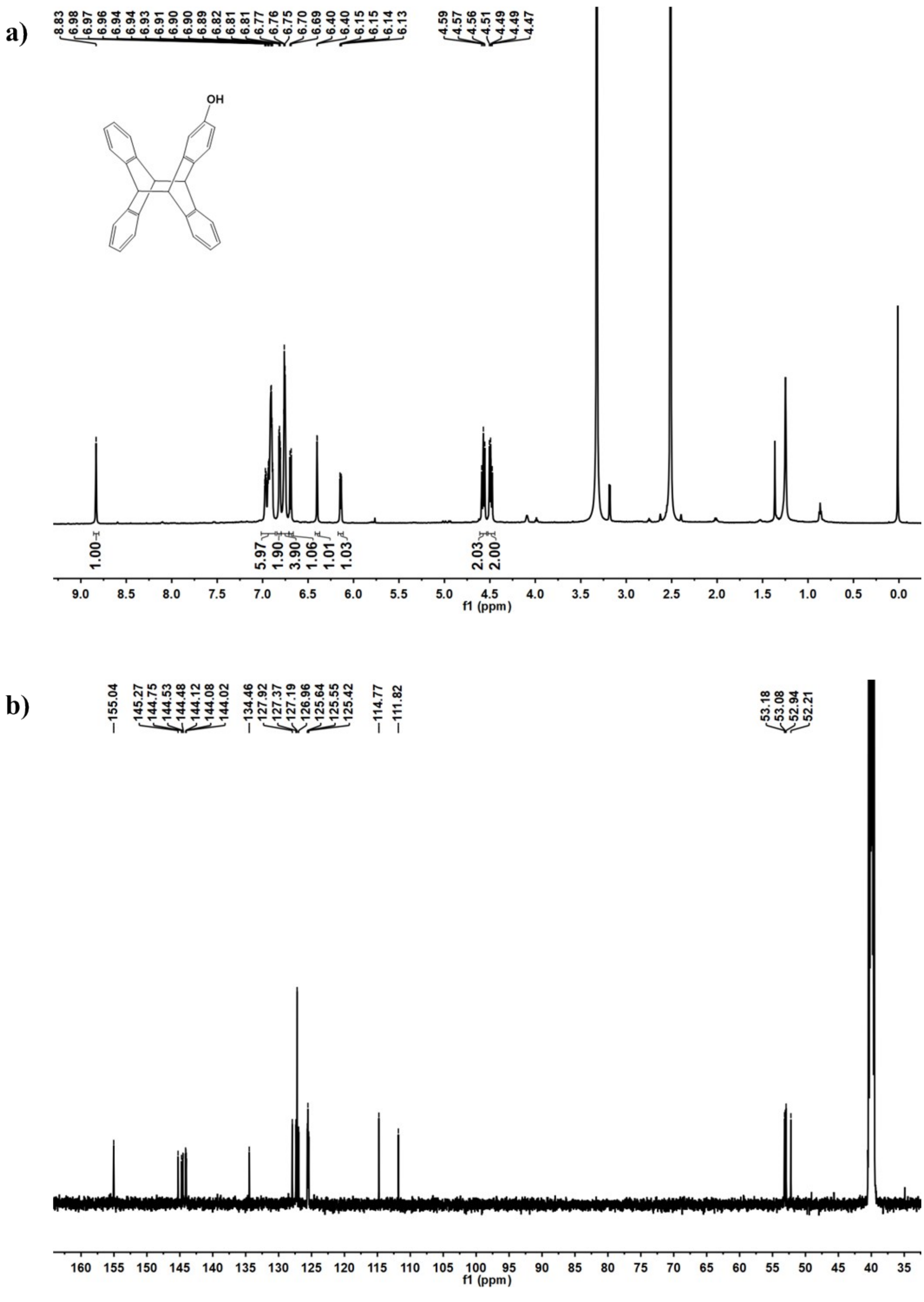

Figure S1. ^{1}H NMR (a) and ^{13}C NMR (b) spectra of 2-hydroxytetraptycene **1** in DMSO-d_6.

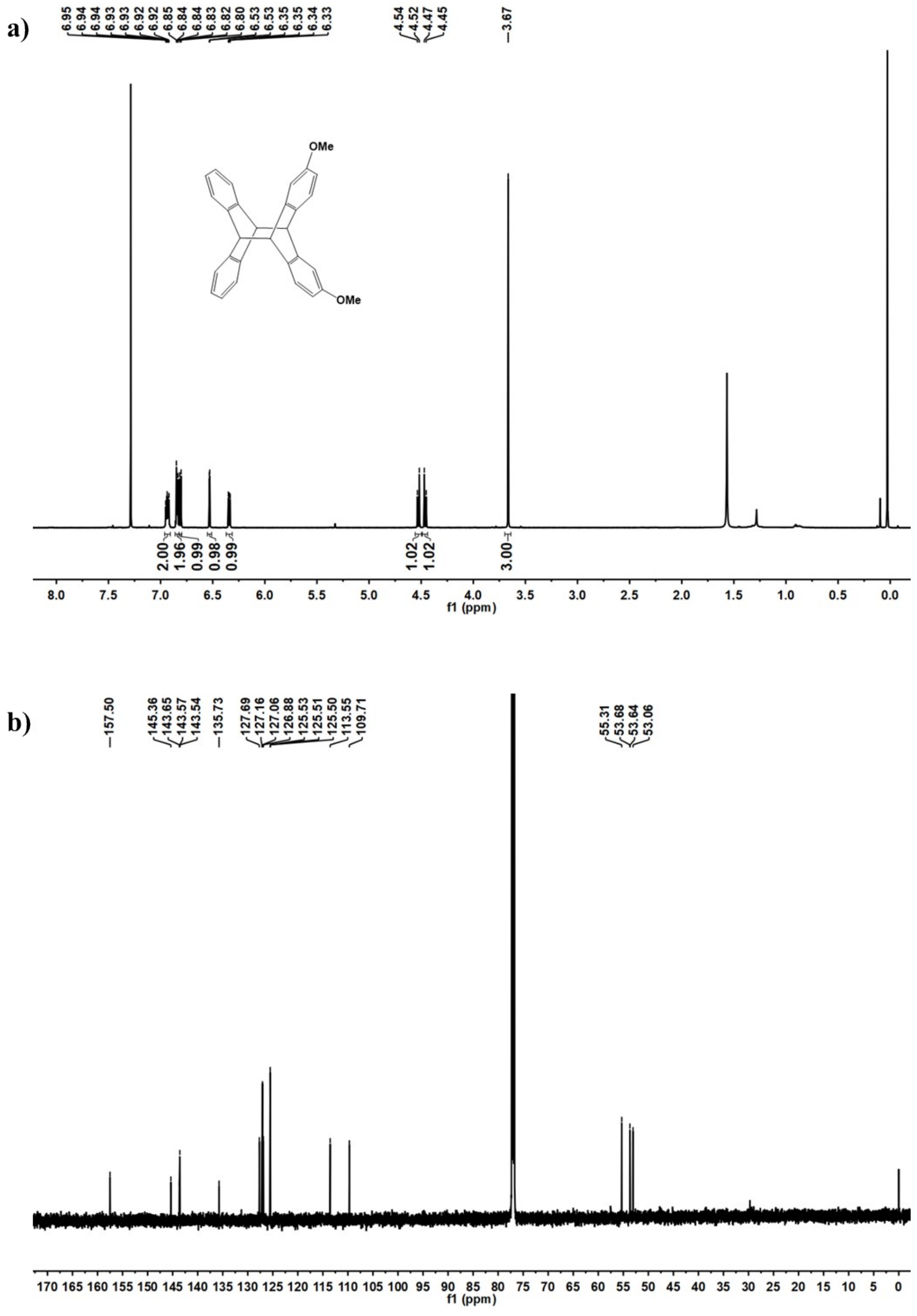

Figure S2. ^{1}H NMR (a) and ^{13}C NMR (b) spectra of 2,6-dimethoxytetraptycene **2** in $CDCl_3$.

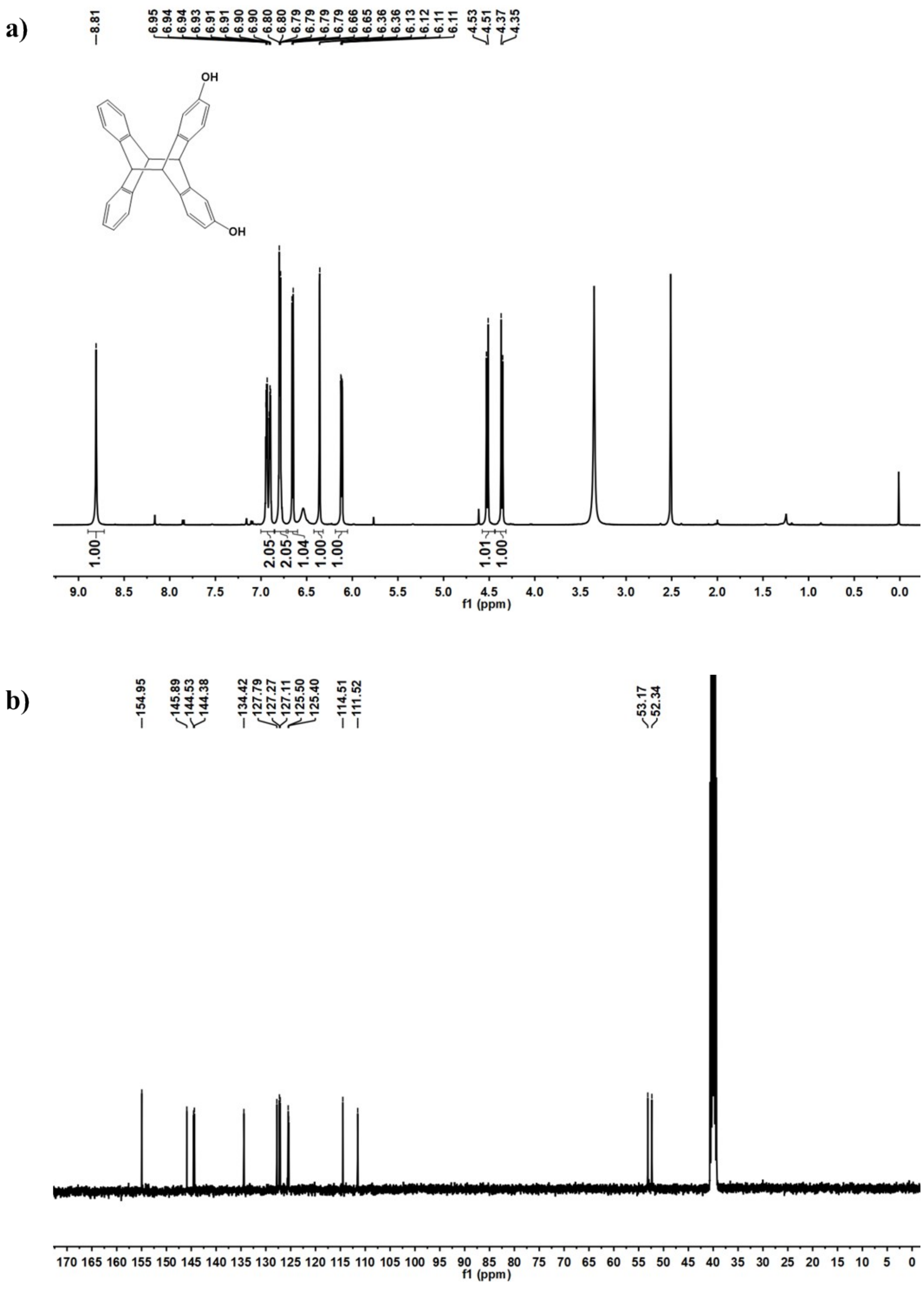

Figure S3. ^{1}H NMR (a) and ^{13}C NMR (b) spectra of 2,6-dihydroxytetraptycene **3** in DMSO-d_6.

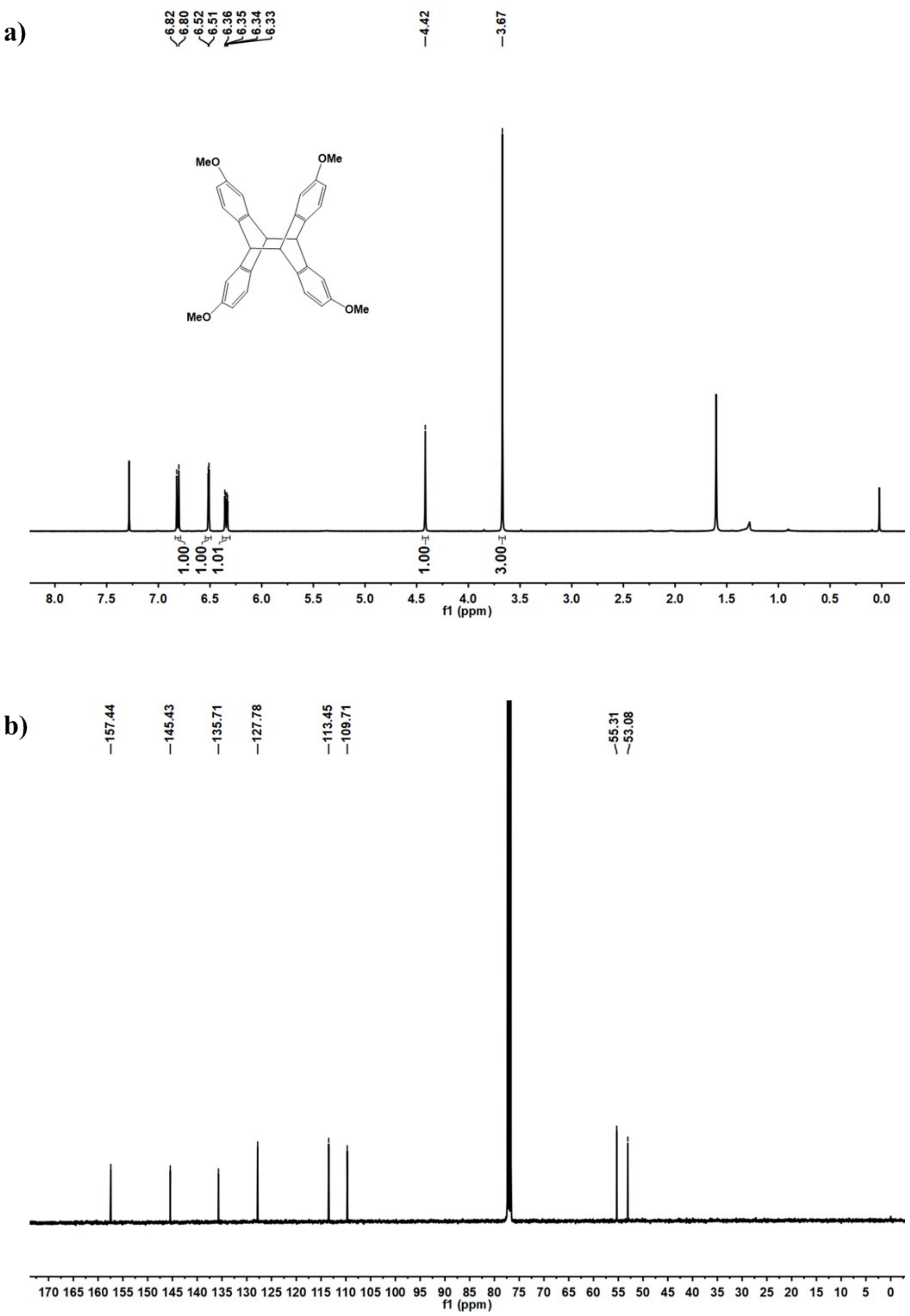

Figure S4. ^{1}H NMR (a) and ^{13}C NMR (b) spectra of 2,2’,6,6’-tetramethoxytetraptycene **4** in $CDCl_3$.

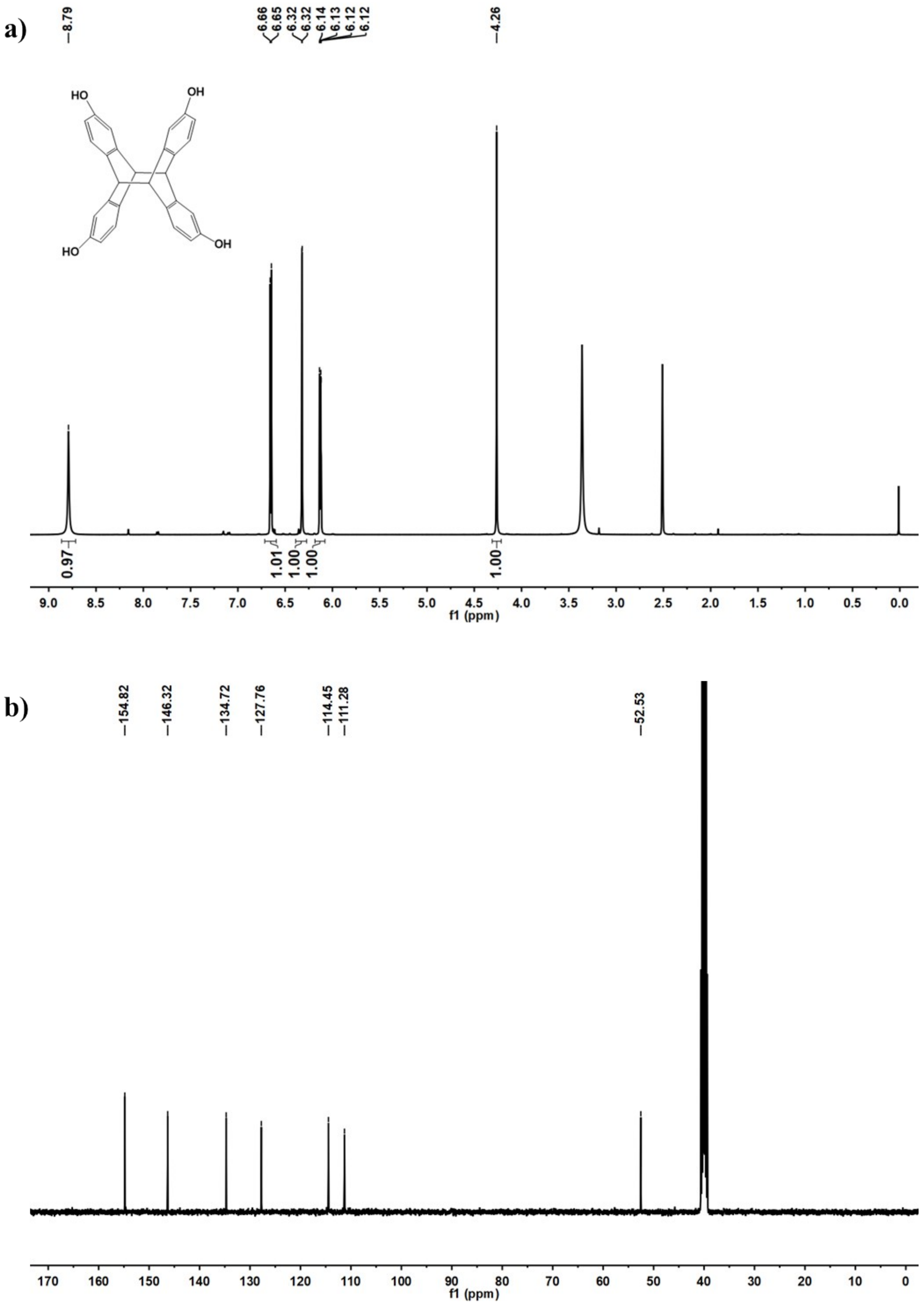

Figure S5. ^{1}H NMR (a) and ^{13}C NMR (b) spectra of 2,2',6,6'-tetrahydroxytetraptycenes **5** in DMSO-d_6.

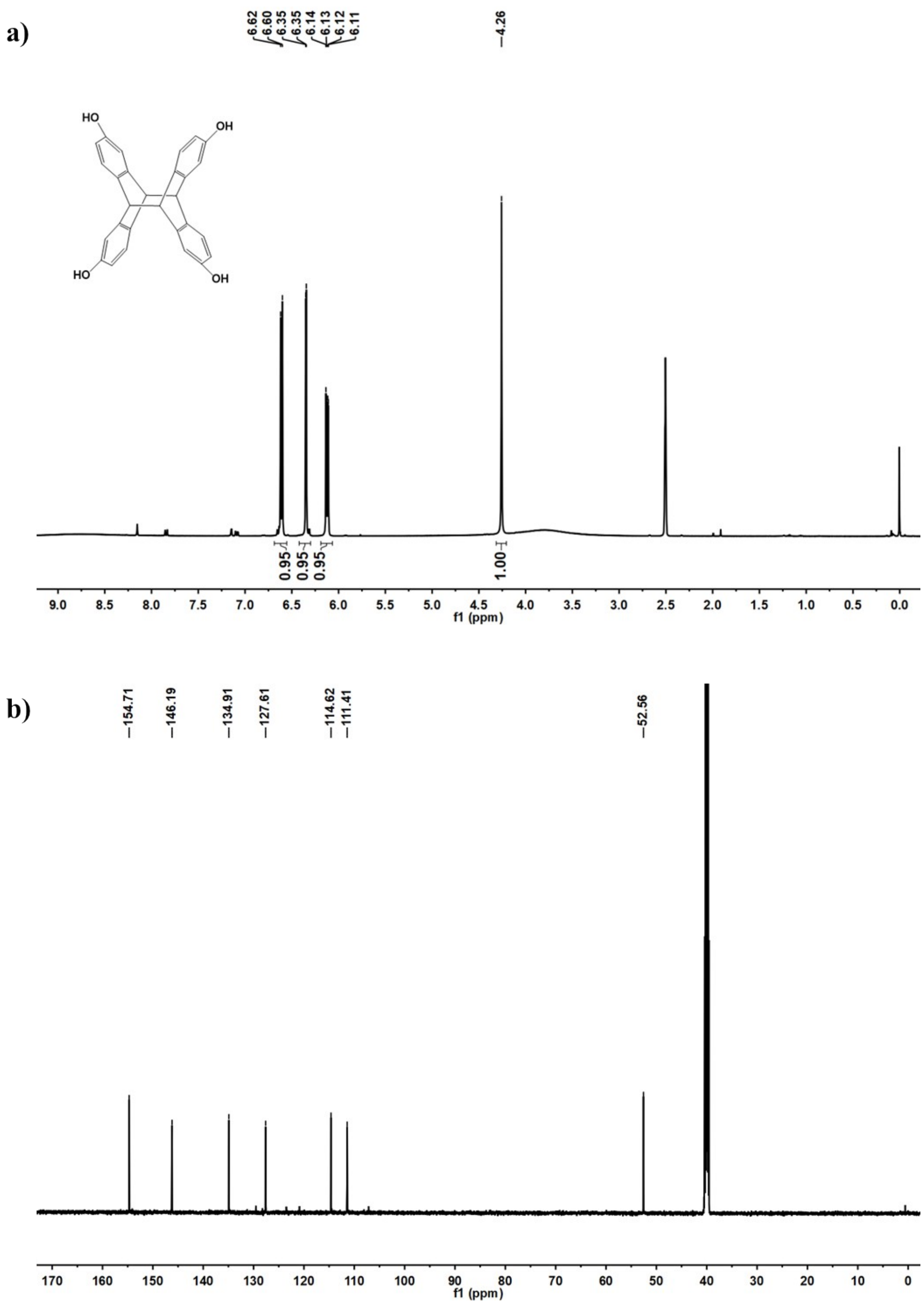

Figure S6. ^{1}H NMR (a) and ^{13}C NMR (b) spectra of 2,2’,6,6’-tetrahydroxytetraptycenes **7** in DMSO-d_6.

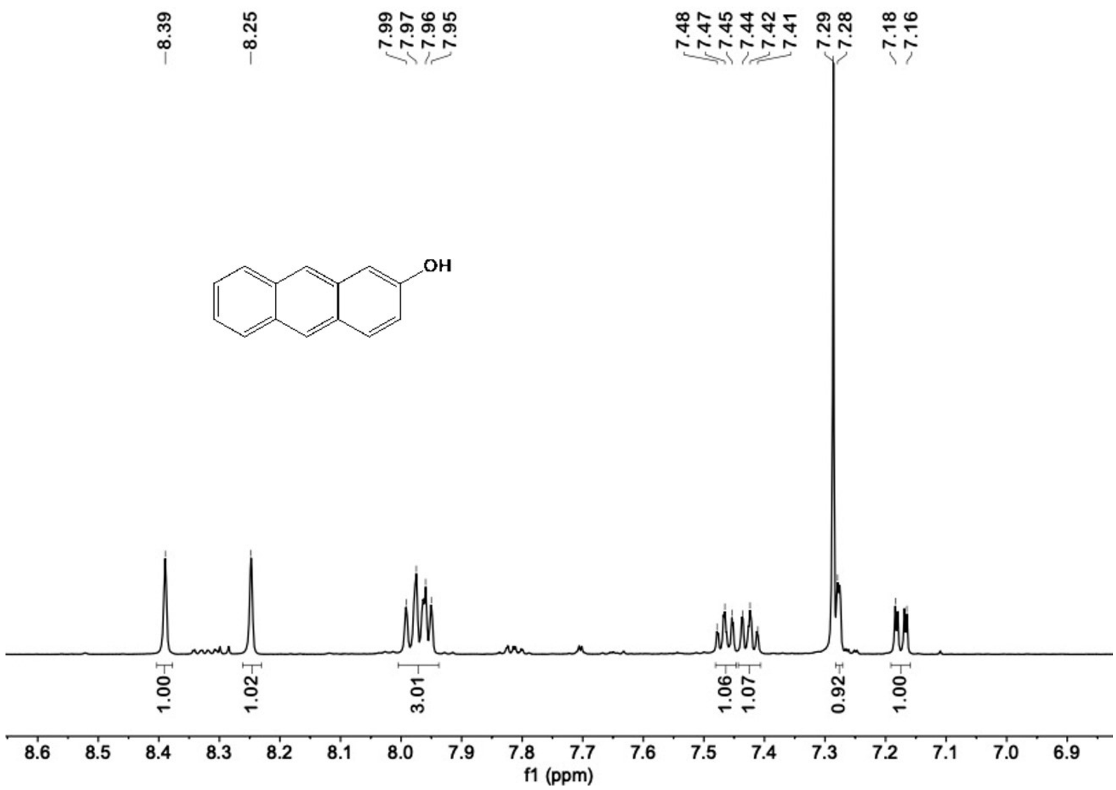

Figure S7. ^{1}H NMR spectra of 2-hydroxyanthracene in $CDCl_3$. ^{1}H NMR (600 MHz, $CDCl_3$): δ 8.3894 (s, 1H), 8.2477 (s, 1H), 8.0050 – 7.9375 (m, 3H), 7.4808-7.4465 (m, 1H), 7.4234 (t, J = 7.4 Hz, 1H), 7.2792 (s, 1H), 7.1743 (d, J = 11.5 Hz, 1H).

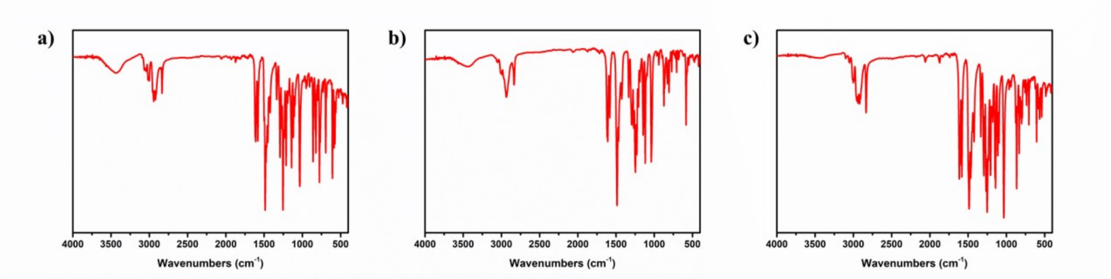

Figure S8. Fourier-transform infrared Spectroscopy of 2,6-dimethoxytetraptycene **2** (a), 2,2',6,6'-tetramethoxytetraptycene **4** (b) and **6** (c).

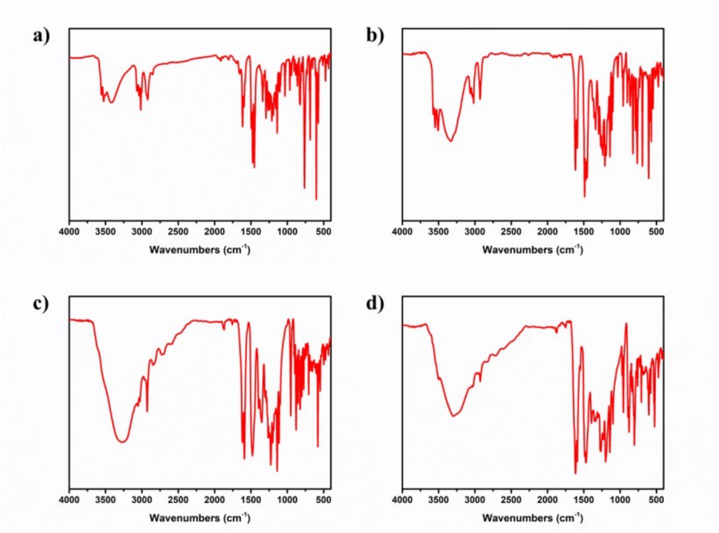

Figure S9. Fourier-transform infrared Spectroscopy of 2-hydroxyanthracene **1** (a), 2,6-dihydroxytetraptycene **3** (b), 2,2’,6,6’-tetrahydroxytetraptycene **5** (c) and **7** (d).

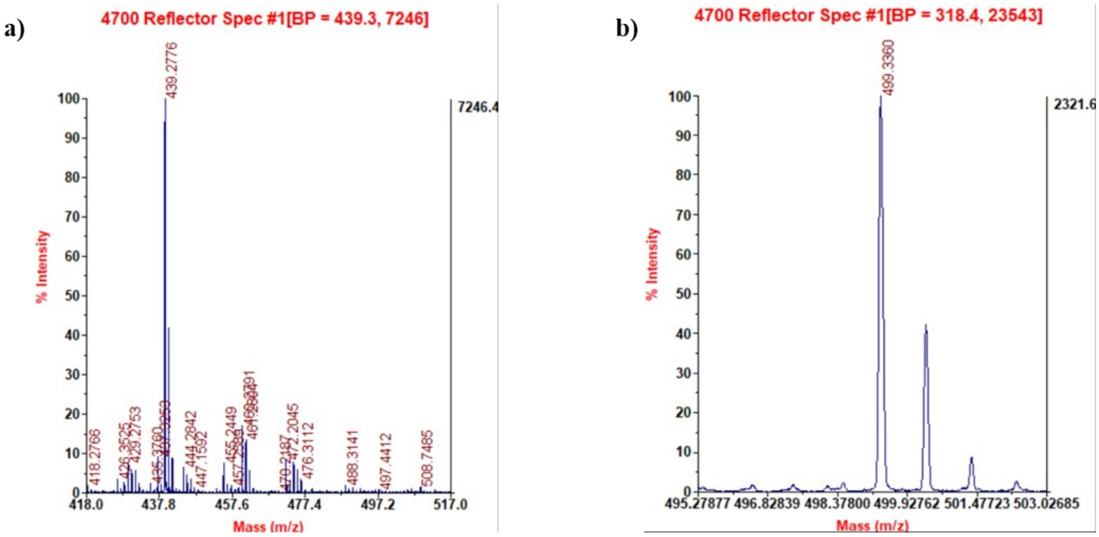

Figure S10. Mass spectrometry of 2,6-dimethoxytetraptycene **2** (a), 2,2’,6,6’-tetramethoxytetraptycene **4** (b).

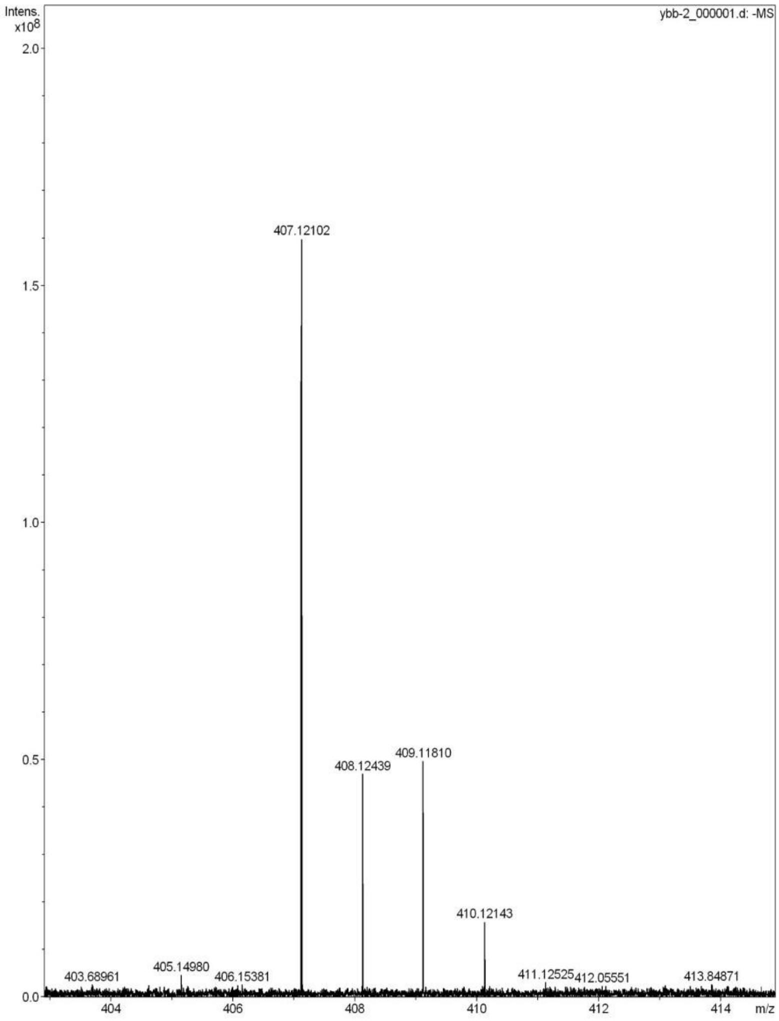

Figure S11. Mass spectrometry of 2-hydroxyanthracene **1**.

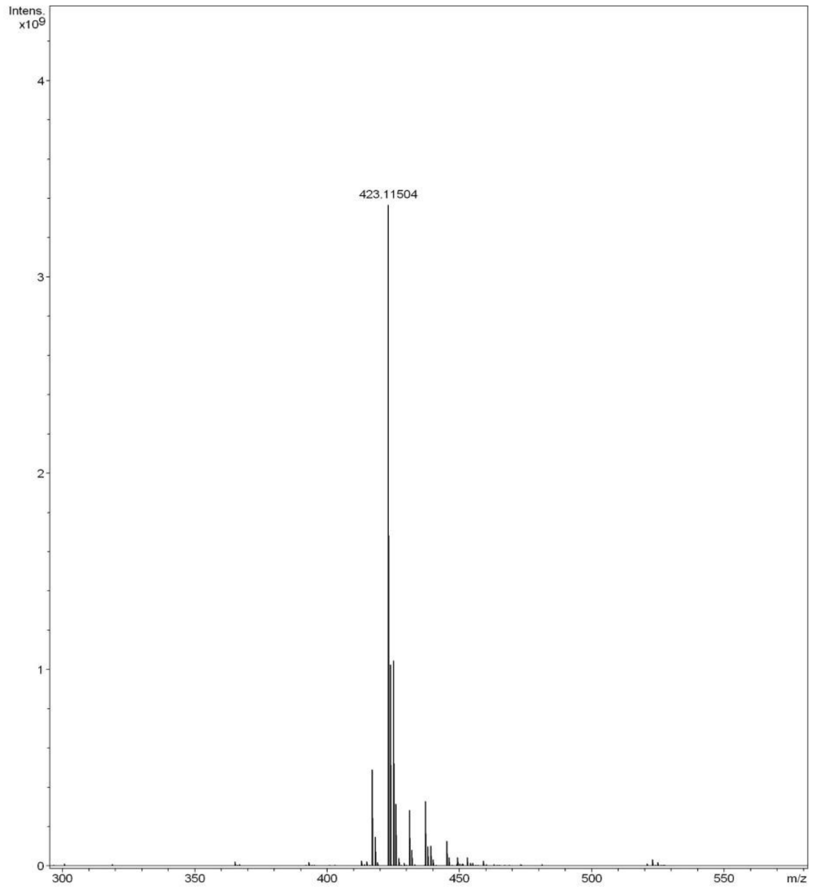

Figure S12. Mass spectrometry of 2,6-dihydroxytetraptycene **3**.

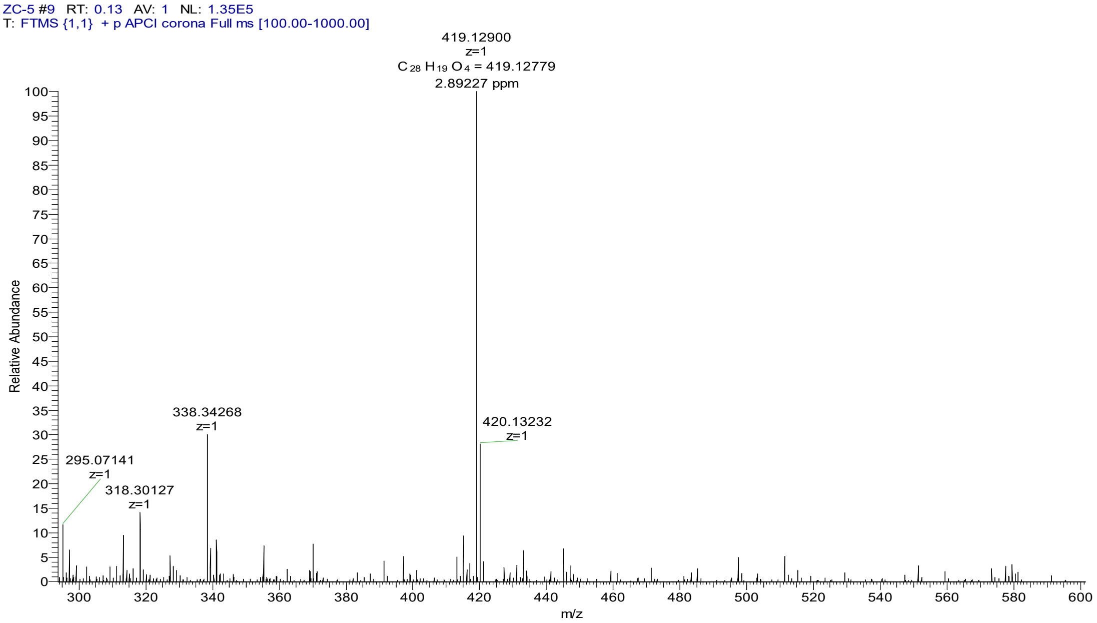

Figure S13. Mass spectrometry of 2,2’,6,6’-tetrahydroxytetraptycene **5**.

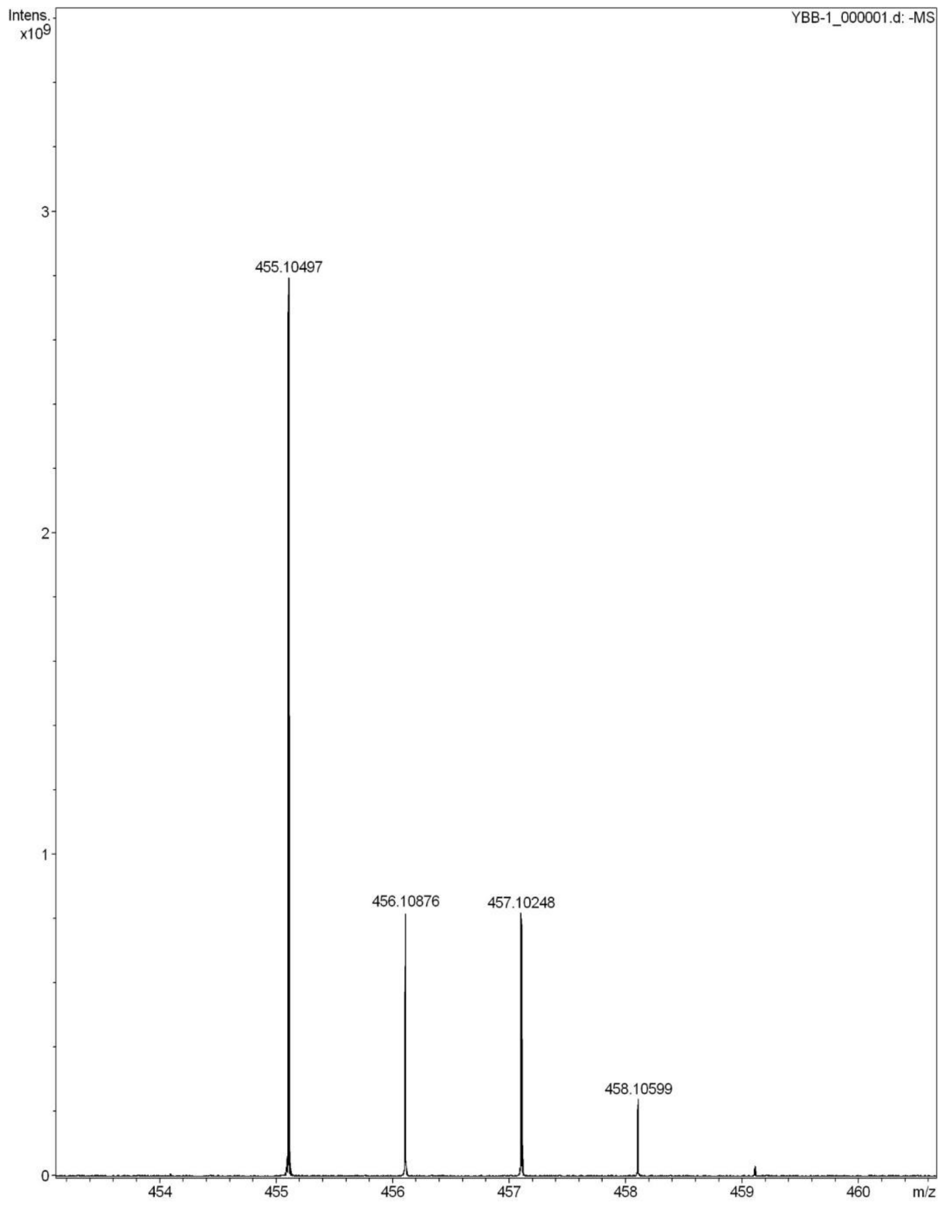

Figure S14. Mass spectrometry of 2,2’,6,6’-tetrahydroxytetraptycene **7**.

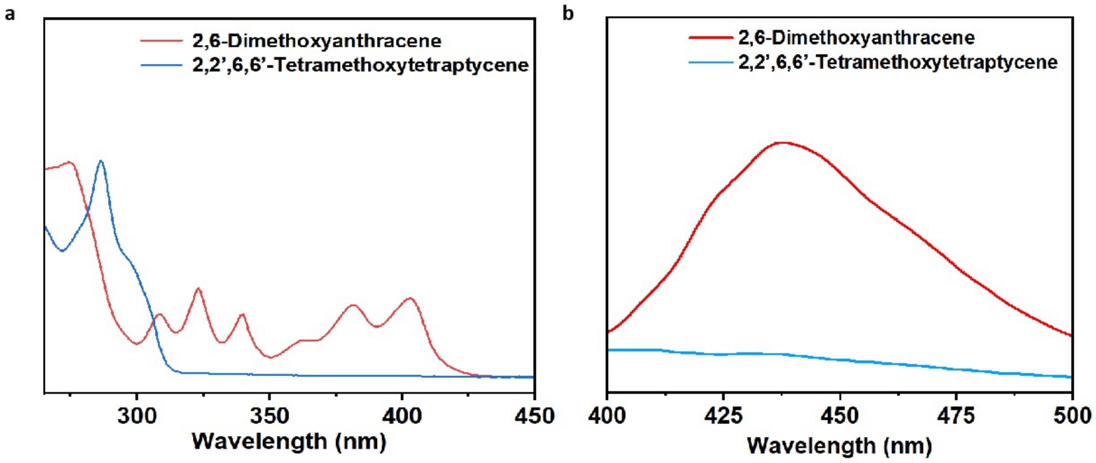

Figure S15. (a) The UV spectra of 2,2',6,6'-tetramethoxytetraptycene (2.0×10^{-6} mol/L, CH_2Cl_2) and 2,6-dimethoxyanthracene (4.2×10^{-6} mol/L, CH_2Cl_2). (b) The fluorescence emission spectra of 2,2',6,6'-tetramethoxytetraptycene (2.0×10^{-8} mol/L, CH_2Cl_2) and 2,6-dimethoxyanthracene (4.2×10^{-8} mol/L, CH_2Cl_2).
